# Time-Efficient High-Resolution Large-Area Nano-Patterning of Silicon Dioxide

**DOI:** 10.3390/mi8010013

**Published:** 2017-01-04

**Authors:** Li Lin, Yiyu Ou, Martin Aagesen, Flemming Jensen, Berit Herstrøm, Haiyan Ou

**Affiliations:** 1 Department of Photonics Engineering, Technical University of Denmark, Ørsteds Plads 340, Kongens Lyngby DK-2800, Denmark; llin@fotonik.dtu.dk (L.L.); yiyo@fotonik.dtu.dk (Y.O.); 2 Gasp Solar ApS, Hovmarken 23, 2640 Hedehusene, Denmark; martin.aagesen@gaspsolar.com; 3 DTU Danchip, Technical University of Denmark, Ørsteds Plads 347, Kongens Lyngby DK-2800, Denmark; fj@danchip.dtu.dk (F.J.); bge@danchip.dtu.dk (B.H.)

**Keywords:** electron-beam lithography, nanoimprint lithography, nano-patterning of silicon dioxide

## Abstract

A nano-patterning approach on silicon dioxide (SiO_2_) material, which could be used for the selective growth of III-V nanowires in photovoltaic applications, is demonstrated. In this process, a silicon (Si) stamp with nanopillar structures was first fabricated using electron-beam lithography (EBL) followed by a dry etching process. Afterwards, the Si stamp was employed in nanoimprint lithography (NIL) assisted with a dry etching process to produce nanoholes on the SiO_2_ layer. The demonstrated approach has advantages such as a high resolution in nanoscale by EBL and good reproducibility by NIL. In addition, high time efficiency can be realized by one-spot electron-beam exposure in the EBL process combined with NIL for mass production. Furthermore, the one-spot exposure enables the scalability of the nanostructures for different application requirements by tuning only the exposure dose. The size variation of the nanostructures resulting from exposure parameters in EBL, the pattern transfer during nanoimprint in NIL, and subsequent etching processes of SiO_2_ were also studied quantitatively. By this method, a hexagonal arranged hole array in SiO_2_ with a hole diameter ranging from 45 to 75 nm and a pitch of 600 nm was demonstrated on a four-inch wafer.

## 1. Introduction

Time-efficient high-resolution large-area nano-patterning of SiO_2_ is crucial for producing cost-effective and high-efficiency tandem GaAsP nanopillars on Si solar cells since the diameters of predefined nanoholes in the SiO_2_ layer determine the diameters of selectively grown GaAsP nanowires [1,2,3]. In addition to this specific application, nano-patterning methods are also extensively employed in the field of light emitting diodes (LEDs) to improve light extraction efficiency [4,5], to enhance the internal quantum efficiency [6,7] and to tune the emission peak wavelength [8,9]. For fabrication of nanostructures, various methods of nano-patterning have been investigated including electron-beam lithography (EBL) [10,11], nanoimprint lithography (NIL) [12,13], nanosphere lithography [14,15,16], and rapid thermal annealing of a thin metal layer [17,18,19]. Nevertheless, these methods are usually limited by either low time efficiency, high process cost, or low resolution. Some other advanced nano-scale techniques like scanning probe oxidation assisted with wet etching [20] and amorphization of silicon by laser irradiation [21] can offer maskless processing and good time efficiency but lower resolution in comparison with that of EBL. Consequently, nowadays, NIL assisted with an EBL-based stamp is still widely used in applications demanding high resolution with a nanofeature size as small as 100 nm [22,23]. However, these papers only focus on the nanoimprint technology, without investigating the low throughput EBL. In this paper, the Si nanopillar stamp fabrication using EBL has been thoroughly explored in terms of exposure mode, dose, and how the Si nanopillar size transfers to the nanohole size and its evolvement in the subsequent processing. The efficiency of the EBL could be significantly increased using one-spot exposure by a more than 4400 times faster writing speed compared to the multi-spot exposure. Furthermore, in comparison with one reported result [23], a patterned area four times larger, a higher structure density with a 40% shorter pitch, and a 50%–55% smaller nanofeature size have been obtained. The final nanofeature hole size has been as small as 45–50 nm with acceptable uniformity, which enables growth of GaAsP nanowire solar cells with diameters around 50 nm for high absorption efficiency.

In this work, a time-efficient high-resolution large-area nano-patterning method of SiO_2_ by NIL using a Si stamp produced by EBL is demonstrated, which possesses further improved time efficiency and structure scalability by the one-spot exposure method, presenting an orderly organized nanopattern in SiO_2_ with the superlatively small size of 45 nm [24]. The impact of exposure dose during one-spot exposure EBL on the Si nanopillar diameter was investigated. In addition, size evolution of nanostructures related to NIL was also studied. In the end, the relation between the diameter of the Si nanopillars and that of the SiO_2_ nanoholes was found in order to enable the fabrication of SiO_2_ nanoholes with different desired diameters. The present results are applicable to various materials or applications.

## 2. Fabrication Method

**Figure 1:**
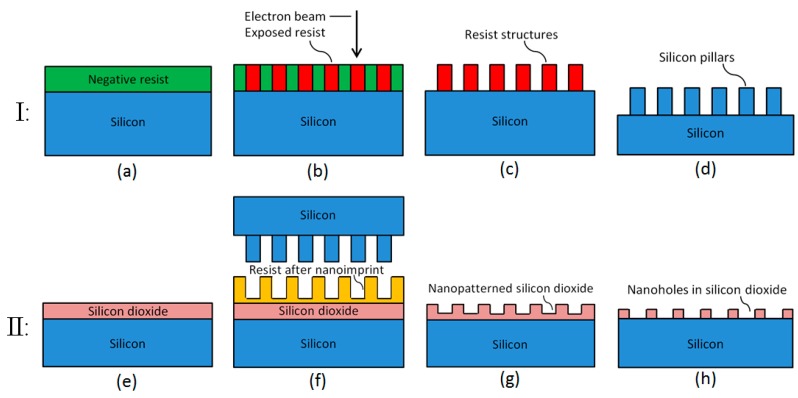
Schematic illustration of the SiO_2_ nanohole fabrication steps: Fabrication of Si stamp (Section I): (**a**) Spin coating of negative resist on Si wafer. (**b**) Electron-beam exposure using negative resist. (**c**) Development to produce resist pillars. (**d**) Si etch by deep reactive-ion etching (DRIE) to produce Si pillars; Fabrication of SiO_2_ nanoholes (Section II): (**e**) Dry oxidation of Si to produce a SiO_2_ layer. (**f**) Spin coating of resist and hot embossing using the Si stamp. (**g**) Removal of residual resist by reactive-ion etching (RIE) followed by SiO_2_ etch by RIE for nano-patterning and subsequent removal of resist. (**h**) SiO_2_ wet etching to expose the Si surface in nanoholes.

The fabrication process of SiO_2_ nanoholes is schematically illustrated in Figure 1, which includes the fabrication of a Si stamp (Section I) and the production of SiO_2_ nanoholes using the Si stamp (Section II). The fabrication process of the Si stamp employs EBL assisted with negative resist for nano-patterning followed by a deep reactive-ion etching (DRIE) process to produce Si pillars ((a)–(d)). Afterwards, the Si stamp was employed in a NIL process for nano-patterning followed by a reactive-ion etching (RIE) process in order to transfer the pattern to the SiO_2_ layer ((e)–(h)).

In the Si stamp fabrication, a Si wafer was first baked at 200 °C to drive out moisture. Afterwards, an 80 nm layer of negative resist mr-EBL 6000 was deposited on the wafer by spin coating (Figure 1a). The nano-patterning of resist was realized by electron beam exposure (Figure 1b) using a JEOL JBX-9500FS EBL system (acceleration voltage = 100 kV, JEOL Ltd., Tokyo, Japan) together with subsequent development (Figure 1c) to produce resist pillars. Next, Si pillars with a height of around 100 nm were produced on the Si wafer by a dry etch process in DRIE (Figure 1d), which functioned as a stamp for a later NIL process. Finally, an FDTS (Cl_3_Si(CH_2_)_2_(CF_2_)_7_CF_3_) layer was deposited using molecular vapor deposition (MVD), which created a molecular film on the stamp surface serving as an anti-stiction coating in the following NIL process.

In order to achieve the designed pattern with a small feature size in EBL, it is critical to optimize three essential parameters: exposure current, shot pitch, and exposure dose. The pattern designed for EBL is a hexagonal arranged square array with a pitch of 600 nm and a side length of 50 nm. The exposure current used is 2 nA and the shot pitch, which is the distance between two neighboring exposure positions of the scanning electron beam, was set to 50 nm, which is also the side length of the squares in the design. Because of the shot pitch which is identical to the size of the square in the hexagonal array, each square was exposed by only one electron beam shot instead of the normally used multiple-spot exposure. In comparison with the normal multiple-spot exposure, one-spot exposure can significantly increase the time efficiency by a more than 4400 times faster writing speed than that of the multi-spot exposure. In the end, it only took 3 h to fabricate the 4″ Si stamp using the one-spot exposure method. In addition, smaller feature sizes can be realized by the one-spot exposure due to the reduced proximity effect. Furthermore, since each square was exposed by a Gaussian electron beam shot, the square in the design can evolve into a roughly round shape after development. Meanwhile, by careful control of the exposure dose, the diameter of the resist nanopillars after development can be effectively tuned, thus generating differently sized resist pillars that will result in Si nanopillars with different diameters after a Si DRIE process. In this work, exposure doses ranging from 10 to 40 µC/cm^2^ were investigated in order to map out the impact of the dose.

In the subsequent SiO_2_ nanohole fabrication, a (111) Si wafer with a 36-nm-thick SiO_2_ layer on the surface, which was formed by dry oxidation (Figure 1e), was spin coated by a 110-nm layer of imprint resist mr-l 7010E, which is a thermoplastic polymer. This (111) Si wafer was employed to realize the perpendicular growth of GaAsP nanowires since, when using the (100) Si wafer, the nanowires tend to have an inclined growth. Afterwards, the nanoimprint was carried out through a hot embossing process using an EVG520HE semi-automated hot embossing system (EV Group, Schärding, Austria) assisted with the previously fabricated Si stamp (Figure 1f). This was followed by the removal of residual resist using RIE, which finally produced the nanohole pattern on the imprint resist layer. Next, SiO_2_ nanoholes were formed by firstly a RIE process with an etch depth of 30 nm in the SiO_2_ layer (Figure 1g). Then, after resist removal, a wet etching process using a 12.5% BHF etchant diluted by a wetting agent and deionized (DI) water with a volume ratio of 1:14 was applied to remove the residual 6 nm SiO_2_ (Figure 1h) and expose the Si surface. In the end, nanoholes with different diameters on 30-nm-thick SiO_2_ were successfully produced on the (111) Si surface.

In fabrication of nanoholes in the SiO_2_ layer by NIL assisted with RIE, the Si pillar diameter can significantly affect the diameter of the final SiO_2_ nanohole. Furthermore, the structure size varies during the different process steps including the nanoimprint in the resist, the removal of residual resist by RIE, the 30 nm etch of SiO_2_ to produce nanoholes by RIE, and the final 6 nm wet etch of SiO_2_ to expose the Si surface underneath. Hence, the diameter of the nanostructures was monitored after each process step to sort out the effect of them separately on the structure size.

## 3. Results and Discussion

**Figure 2:**
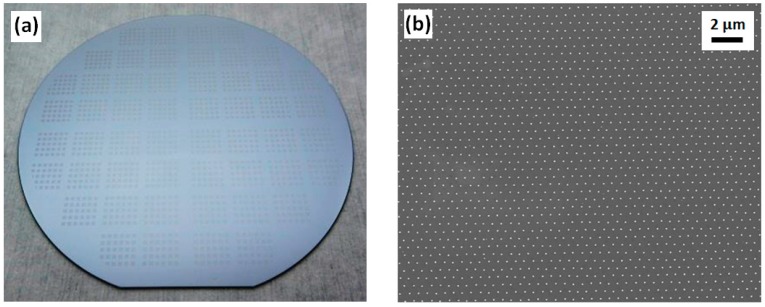
Images of a four-inch Si stamp after electron-beam lithography (EBL), DRIE, and removal of resist: (**a**) Image of the Si stamp taken by camera. (**b**) Top-view scanning electron microscopy (SEM) image of the Si nanopillars related to the exposure dose of 40 µC/cm^2^.

**Figure 3:**
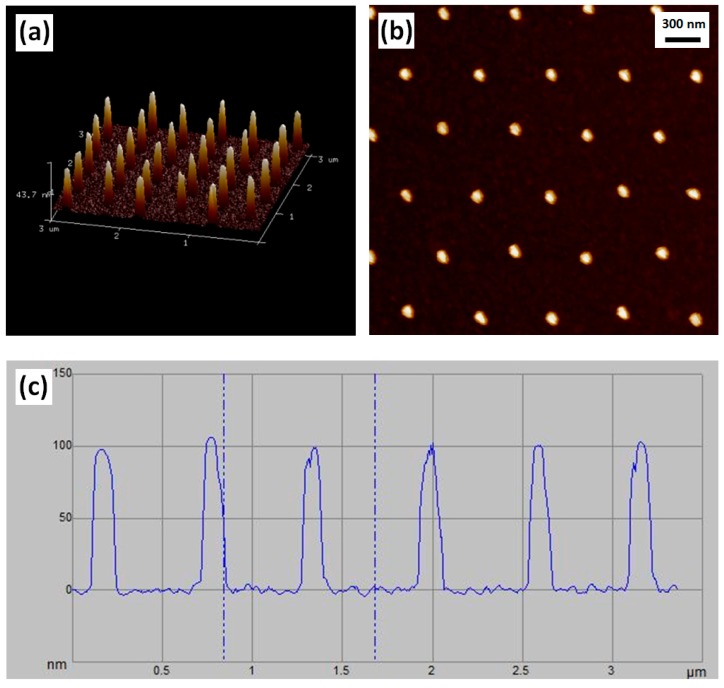
Atomic force microscopy (AFM) images of Si nanopillars related to the exposure dose of 40 µC/cm^2^: (**a**) 3-D image of the Si nanopillars. (**b**) Top-view image of the Si nanopillars. (**c**) Curve of the scanned pillar height.

In the Si stamp fabrication using one-spot electro-beam exposure and negative resist mr-EBL 6000.1, doses ranging from 10 to 40 µC/cm^2^ under an exposure current of 2 nA were employed. After the dry etch process, a hexagonal array consisting of Si nanopillars with a pitch of 600 nm was successfully fabricated showing that the Si nanopillar diameter depends significantly on the exposure dose. Figure 2 presents a picture of the four-inch Si stamp (Figure 2a) and a scanning electron microscopy (SEM, ZEISS, Oberkochen, Germany) image of Si nanopillars on the stamp (Figure 2b). Atomic force microscopy (AFM, Bruker, Billerica, MA, USA) images of Si nanopillars at the exposure dose of 40 µC/cm^2^ are shown in Figure 3, demonstrating a 3-D view of the nanopillars (Figure 3a), a top view of the nanopillars (Figure 3b), and a measured curve showing a nanopillar height of around 100 nm (Figure 3c).

**Figure 4:**
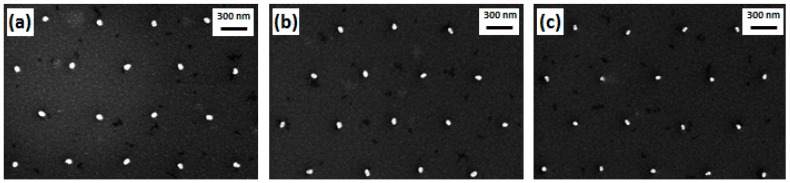
Top-view SEM images of Si nanopillars with different diameters resulting from corresponding different doses: (**a**) 40 µC/cm^2^; (**b**) 34 µC/cm^2^; (**c**) 28 µC/cm^2^.

**Figure 5:**
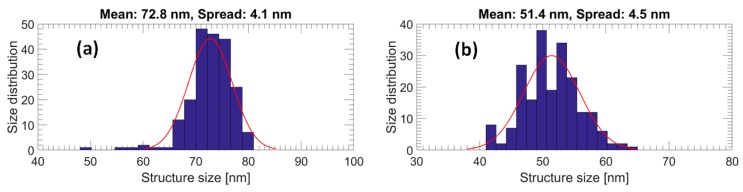
Diameter distribution measured from top-view SEM images of Si nanopillars on Si stamp exposed by different doses: (**a**) 40 µC/cm^2^; (**b**) 28 µC/cm^2^.

Generally, a higher dose results in a larger pillar diameter and a higher pillar yield. To be more precise, doses from 26 to 40 µC/cm^2^ result in Si nanopillars with diameters from 40 to 70 nm. Si pillars related to doses lower than 28 µC/cm^2^ have a considerably low yield. Figure 4 shows top-view SEM images of Si nanopillars related to exposure doses of 40 µC/cm^2^ (Figure 4a), 34 µC/cm^2^ (Figure 4b), and 28 µC/cm^2^ (Figure 4c), respectively. As a consequence of having a variety of resist pillar diameters on the stamp, there will be a local difference in the Si etch rate due to the aspect ratio dependent etching (ARDE). In the present work, this means that the Si pillar height ranges from 85 to 105 nm as the exposure dose is varied from 26 to 40 µC/cm^2^. Size distributions of nanopillars exposed by doses of 40 and 28 µC/cm^2^, respectively, are shown in Figure 5 presenting Gaussian distributions. The size distribution was analyzed by importing the SEM top-view picture into a Matlab program (R2015b, MathWorks, Natick, MA, USA) which can convert the picture to a binary one with a structure color in white and background color in black and extract the pillar diameter from the area in white. A total area of 10 µm × 6 µm on the stamp containing around 200 Si nanopillars was used to obtain the distribution. The mean diameters are 72.8 and 51.4 nm for Si nanopillars related to 40 and 28 µC/cm^2^, respectively, with diameter variations of around 4 nm. It is shown that a lower exposure dose in EBL can result in a smaller structure size.

**Figure 6:**
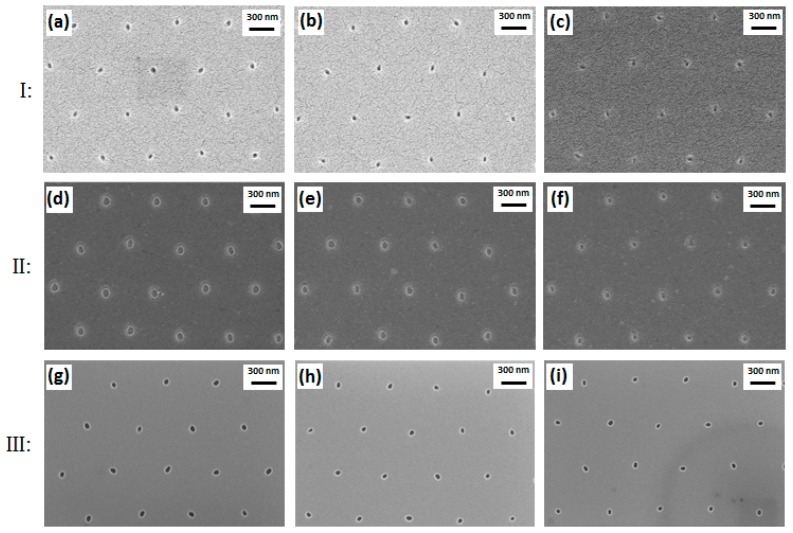
Top-view SEM images of nanoholes with different diameters resulting from corresponding nanopillars realized by different doses: Nanoholes in resist after nanoimprint (Section I): (**a**) 40 µC/cm^2^; (**b**) 34 µC/cm^2^; (**c**) 28 µC/cm^2^. Nanoholes in resist after residual resist removal by RIE (Section II): (**d**) 40 µC/cm^2^; (**e**) 34 µC/cm^2^; (**f**) 28 µC/cm^2^. Nanoholes in SiO_2_ produced by combined RIE and wet etching (Section III): (**g**) 40 µC/cm^2^; (**h**) 34 µC/cm^2^; (**i**) 28 µC/cm^2^.

**Figure 7:**
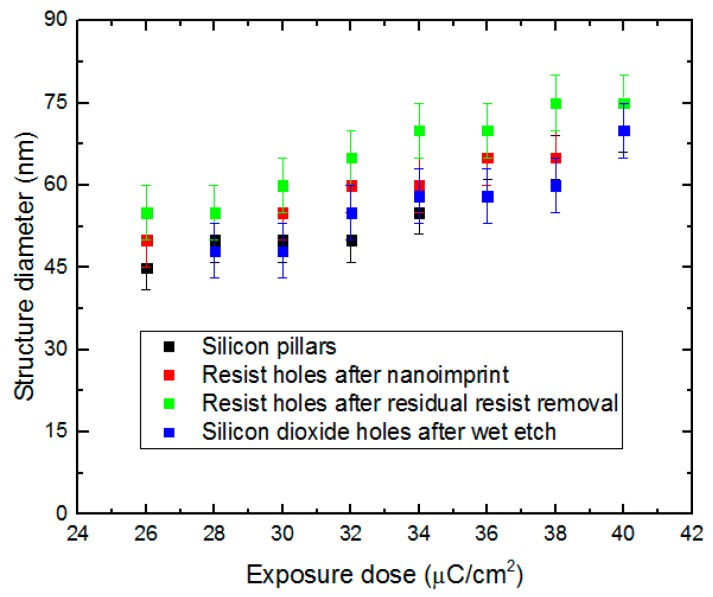
Nanostructure diameters after different fabrication steps.

After nanoimprint assisted with the Si stamp with nanopillars, nanoholes were successfully produced in the resist layer. The nanohole diameter is determined by the diameter of the corresponding Si nanopillar. In comparison with the corresponding Si nanopillar diameters of the stamp, holes produced in the resist layer were increased by less than 5 nm. Figure 6, Section I, shows top-view SEM images of nanoholes in the resist after nanoimprint. Furthermore, the etch of the residual resist by RIE gave a further increase of less than 5 nm. Consequently, there would be a total increase of less than 10 nm in comparison with the corresponding Si nanopillar diameters. Top-view SEM images of nanoholes in resist after the RIE process of the residual resist are shown in Figure 6, Section II. The RIE process of SiO_2_ successfully transferred the nanopattern of imprint resist to the SiO_2_ layer. After the final wet etch of SiO_2_, the hole diameters in SiO_2_ were decreased by 5 to 10 nm in comparison with the hole diameters after RIE etch of the residual imprint resist. Figure 6, Section III, shows top-view SEM images of nanoholes in SiO_2_ after RIE and wet etching processes. Although the dimension difference in nanometer is too tiny to be clearly observed, the dimension change resulting from Si pillars exposed by different doses can still be inspected from these figures. For a better comparison of the structure sizes, Figure 7 shows the mean values and size variations of Si nanopillar diameters related to different electron-beam exposure doses and the hole diameters after nanoimprint, the RIE of residual imprint resist, and the RIE and wet etching of SiO_2_, respectively. The SiO_2_ nanoholes related to the exposure doses lower than 28 µC/cm^2^ cannot be clearly observed due to insufficient pillar height by ARDE. In addition, as shown in Figure 7, larger exposure doses in EBL lead to larger structure diameters. Regarding the Si nanopillars, around 200 of these were measured within one SEM image under a magnification of 20 KX, while approximately 20 nanoholes in resist and SiO_2_ were measured within one SEM image under a magnification of 100 KX due to bad electron conductivity of resist and SiO_2_. In the latter cases (resist and SiO_2_), it is difficult to obtain SEM images with good quality due to the severe charging effect of the nonconductive materials. Hence, nanoholes in these materials were only inspected under high magnification with a limited number of structures. However, in addition to the nanohole size measurement, the size variations of Si nanopillars shown in Figure 5 also indicate acceptable structure size uniformity.

## 4. Conclusions

In summary, a time-efficient high-resolution large-area nano-patterning method of SiO_2_ by NIL assisted with a reusable Si stamp produced by EBL has been demonstrated. The acceptable Si stamp degradation realizes improved time efficiency for mass production by NIL. In addition, further improved time efficiency and structure scalability are obtained by the one-spot exposure method in EBL presenting an orderly organized nanopattern in SiO_2_ on a four-inch wafer. The one-spot exposure process increases the time efficiency of EBL by a more than 4400 times faster writing speed than that of the multi-spot exposure. Furthermore, smaller feature sizes can be realized by this method due to the reduced proximity effect by one-spot exposure. Moreover, it is found that, by precisely altering the exposure dose, the SiO_2_ nanohole diameter is tunable. Si nanopillars on the stamp with a tunable diameter ranging from 40 to 70 nm were produced by EBL with exposure doses ranging from 26 to 40 µC/cm^2^. For exposure doses lower than 28 µC/cm^2^, the nanohole yield was significantly degraded. In the subsequent NIL producing SiO_2_ nanoholes with a depth of 30 nm, a strict control of pattern transfer is critical, including nanoimprint, removal of residual resist, and RIE and wet etching of SiO_2_. In the end, nanoholes in SiO_2_ with a tunable diameter ranging from 45 to 75 nm were produced depending on the corresponding Si nanopillar dimension. The diameter difference between the final SiO_2_ nanoholes and the Si nanopillars is within 5 nm. The diameters of nanoholes in SiO_2_ as small as 45–50 nm present a 50%–55% size reduction in comparison with the diameter of 100 nm stated in the referenced past work. We find the demonstrated nano-patterning method of orderly organized nanostructures very promising as a highly generic and cost-efficient technique for a large variety of applications.
